# Serum Tau Species in Progressive Supranuclear Palsy: A Pilot Study

**DOI:** 10.3390/diagnostics14232746

**Published:** 2024-12-05

**Authors:** Costanza Maria Cristiani, Luana Scaramuzzino, Elvira Immacolata Parrotta, Giovanni Cuda, Aldo Quattrone, Andrea Quattrone

**Affiliations:** 1 Neuroscience Research Center, Department of Medical and Surgical Sciences, University “Magna Graecia”, 88100 Catanzaro, Italy; 2 Institute of Molecular Biology, Department of Medical and Surgical Sciences, University “Magna Graecia”, 88100 Catanzaro, Italy; parrotta@unicz.it; 3 Research Centre for Advanced Biochemistry and Molecular Biology, Department of Clinical and Experimental Medicine, University “Magna Graecia”, 88100 Catanzaro, Italy; cuda@unicz.it; 4 Institute of Neurology, Department of Medical and Surgical Sciences, University “Magna Graecia”, 88100 Catanzaro, Italy

**Keywords:** progressive supranuclear palsy, Parkinson’s disease, ELISA, p-tau396, serum

## Abstract

Background/Objectives: Progressive Supranuclear Palsy (PSP) is a tauopathy showing a marked symptoms overlap with Parkinson’s Disease (PD). PSP pathology suggests that tau protein might represent a valuable biomarker to distinguish between the two diseases. Here, we investigated the presence and diagnostic value of six different tau species (total tau, 4R-tau isoform, tau aggregates, p-tau202, p-tau231 and p-tau396) in serum from 13 PSP and 13 PD patients and 12 healthy controls (HCs). Methods: ELISA commercial kits were employed to assess all the tau species except for t-tau, which was assessed by a single molecule array (SIMOA)-based commercial kit. Possible correlations between tau species and biological and clinical features of our cohorts were also evaluated. Results: Among the six tau species tested, only p-tau396 was detectable in serum. Concentration of p-tau396 was significantly higher in both PSP and PD groups compared to HC, but PSP and PD patients showed largely overlapping values. Moreover, serum concentration of p-tau396 strongly correlated with disease severity in PSP and not in PD. Conclusions: Overall, we identified serum p-tau396 as the most expressed phosphorylated tau species in serum and as a potential tool for assessing PSP clinical staging. Moreover, we demonstrated that other p-tau species may be present at too low concentrations in serum to be detected by ELISA, suggesting that future work should focus on other biological matrices.

## 1. Introduction

Human tau is a microtubule-associated protein playing a pivotal role in stabilizing microtubules. Within the central nervous system (CNS), tau is predominantly expressed by neurons and to a minor extent in astrocytes and oligodendrocytes. In neurons, tau protein is preferentially located in the axons, where it regulates axonal transport and outgrowth [1]. Based on function and composition, the aminoacidic sequence of the tau protein can be subdivided into four domains: the projection domain at the N-terminal, the proline-rich domain, the microtubule-binding (MTB) domain and the C-terminal domain [1]. In the adult brain, tau exists in six different isoforms due to alternative splicing of exons 2, 3 and 10 of the *MAPT* gene. Particularly, exons 2 and 3 code for an insert at the N-terminus, so the insert can either be absent (0N) or repeated one (1N) or two (2N) times. On the other hand, exon 10 codes for an additional MTB sequence, therefore the MTB domain can be composed of three (3R) or four (4R) MTB repetitions. Accordingly, 4R tau shows a 40-fold higher affinity for microtubules compared to 3R isoform [2].

In its native conformation, tau is a monomeric, unfolded, highly hydrophilic protein. Tau conformation and function, however, are regulated by several types of post-translational modifications, with phosphorylation as the most well-known modification. By adding negative charges, phosphorylation reduces tau affinity for microtubules, which in turn allows a rapid regulation of microtubule dynamics. Indeed, 85 putative phosphorylation sites have been identified within the tau sequence. Hyperphosphorylation has been identified as a key molecular mechanism determining tau pathology in the brain, although the precise residues involved in this process are still unknown [3]. Hyperphosphorylated tau monomers not only detach from microtubules impairing cytoskeleton dynamics, but also tend to self-assemble into small oligomers and then in fibrils to form neurofibrillary tangles (NFTs), which appear to be neurotoxic. NFTs represent the histopathological mark of many neurodegenerative disorders collectively known as tauopathies [1].

Progressive Supranuclear Palsy (PSP) is a tauopathy characterized by the presence of NFTs composed of 4R tau isoforms, oligodendroglial coiled bodies, tufted astrocytes, gliosis and neuronal loss [4]. Clinically, PSP belongs to parkinsonism, characterized by postural and gait impairment, rigidity and bradykinesia [5,6]. Compared to Parkinson’s Disease (PD), which is a synucleinopathy, PSP typically shows a more aggressive phenotype with additional specific symptoms such as vertical supranuclear gaze palsy or slowness of vertical saccades [6]. However, these signs can occur late in the course of the disease, and PSP and PD display marked symptom overlap, especially at the early stages, which in turn causes a high rate of misdiagnosis [5,6,7]. For this reason, the identification of a diagnostic biomarker aiding the physicians in clinical practice is urgently needed. In view of its role in PSP pathology, tau protein has been preliminarily assessed as a potential biomarker to distinguish PSP from PD. Among the many tau species (4R, 3R, several phosphorylated forms) identified in PSP brains, previous studies in parkinsonian syndromes selectively investigated t-tau and p-tau181 levels in CSF, with a few reports also in peripheral fluids.

The results of individual studies were conflicting, with some authors reporting increased CSF t-tau in PSP [8,9] than in PD and HC and others reporting similar values across PSP, PD and HC [10,11]. Notably, a recent work demonstrated that CSF t-tau does not correlate with either inflammation or brain atrophy, two common features associated with PSP [12], thus suggesting that t-tau might not actually reflect ongoing systemic or neurological impairment in PSP. Regarding p-tau181, most studies found similar values in CSF across parkinsonian syndromes and controls [8,10,11,13] but elevated in patients with Alzheimer’s disease [10]. A large meta-analysis reported reduced CSF t-tau and p-tau181 in PD than in HC, but it did not include PSP patients [14].

Recent efforts evaluated the possibility of developing a simple and poorly invasive diagnostic test using more accessible fluids such as blood-derived matrices. In this context, some studies investigated t-tau and p-tau181 in plasma, but the findings were conflicting and heterogeneous [15,16,17,18,19,20,21], and only one study assessed p-tau181 in serum, showing no differences among PSP, PD and HC [22]. On the other hand, other species of tau identified in PSP brains, such as 4R isoform and aggregates [4] as well as other phosphorylated species [23,24,25,26,27,28] have been scarcely assessed in the periphery. To date, only one study focused on plasma p-tau231 as a biomarker to discriminate between PD and HC [29], but no study investigated this or other tau species in plasma or serum in parkinsonian syndromes, highlighting the need for further work in this largely unexplored field.

Here, we performed a preliminary study assessing six different tau species (total, 4R, aggregated, p-tau202, p-tau231 and p-tau396) in the serum in PD and PSP patients with the aim of identifying specific diagnostic biomarkers easily assessable in clinical routine.

## 2. Materials and Methods

### 2.1. Patients

A total of 13 PD and 13 PSP patients fulfilling the MDS diagnostic criteria [5,6] were recruited at the Movement Disorder Center of the Magna Graecia University of Catanzaro. Of the 13 PSP patients, 11 were diagnosed with probable PSP-Richardson’s Syndrome (PSP-RS) and 2 with probable PSP-Parkinsonism (PSP-P). All patients underwent a neurological examination with movement disorder specialists, a 3T brain MRI with a recently described protocol [30] and a 123I-FP-CIT-SPECT (DaTscan). The neurological examination included the MDS-Unified Parkinson’s Disease Rating Scale part III (MDS-UPDRS-III) [31] and Hoehn and Yahr (HY) rating scales in practical off-state to score disease severity. PSP patients were further evaluated using the PSP rating scale [32]. Exclusion criteria for both the patient groups included: clinical features suggestive of other diseases, MRI signs suggestive of normal pressure hydrocephalus or normal striatal uptake on 123I-FP-CIT-SPECT. Twelve age- and sex-matched subjects without any psychiatric or neurological disorders and without close relatives affected by neurodegenerative diseases were recruited as HCs. The study was performed according to The Declaration of Helsinki and approved by the Calabria Region Ethics Committee. All the involved subjects gave written informed consent for participation in the study and the use of their medical records for research purposes.

### 2.2. Serum Collection and Biomarkers Assessment

Serum samples from each subject were collected in BD Vacutainer^TM^ SST^TM^ Serum Separation Tubes (BD, Franklin Lakes, NJ, USA) between 9 a.m. and 12 p.m. and processed within 30 min from withdrawal. Samples were centrifuged at 3000 rpm for 10 min at 4 °C, aliquoted and stored at −20 °C until use. For the biomarkers assessment, serum aliquots were then thawed overnight at 4 °C, mixed thoroughly and centrifuged at 2200 rpm for 15 min.

Serum p-tau202, p-tau231, p-tau396, 4R isoform and tau aggregates were evaluated by specific commercial ELISA kits. In detail, kits for p-tau202 (MBS7616840), p-tau231 (MBS7616725) and p-tau396 (MBS7269317) were purchased from MyBioSource (San Diego, CA, USA); kit for 4R isoform (ab309282) was purchased from Abcam (Cambridge, UK); and kit for tau aggregates (847-0104000116) was purchased from Roboscreen GmbH (Leipzig, Germany). All the assays were read on a Varioskan™ LUX multimode microplate reader (Thermo Fisher Scientific, Waltham, MA, USA).

On the other hand, total tau was measured by ultrasensitive single molecule array (SIMOA) technology (101552, Simoa^®^ TAu v2.0, Quanterix, Billerica, MA, USA) on a fully automated Quanterix HD-X™ Automated Immunoassay Analyzer.

To assess tau aggregates, 4R isoform, p-tau202 and p-tau231 undiluted serum was used, while for p-tau396, evaluation samples were diluted 1:3 in PBS 1X. For t-tau assessment, serum was diluted 1:4 according to the manufacturer’s protocol. All the assays were performed in duplicate.

### 2.3. Statistical Analysis

The Shapiro−Wilk test was applied to evaluate normal distribution of continuous variables. Differences in age and p-tau396 serum concentration between all the groups were assessed by ANOVA, followed by Tukey’s LSD post hoc test; differences in disease duration and MDS-UPDRS-III between patient groups were assessed by Student’s t-test while for differences in HY Staging Scale the Mann−Whitney test was used. Chi-squared test exact was used to assess differences in sex distribution between groups. A *p*-value < 0.05 was considered as significant for all the analyses. All statistical analyses were performed by using IBM SPSS v29.0.1.0 (Armonk, NY, USA).

## 3. Results

### 3.1. Differences Between Groups

We investigated a cohort including 13 PSP and 13 PD patients as well as 12 healthy controls (HCs), whose demographic and clinical features are reported in Table 1. There were no differences in sex and age distribution among groups. Since PSP is a more aggressive condition [6], PSP patients showed higher HY and MDS-UPDRS-III scores than PD, reflecting higher clinical severity.

**Figure 1:**
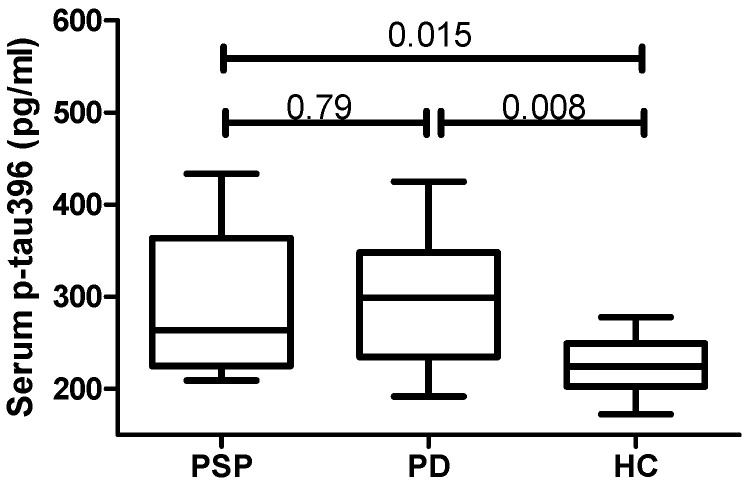
Serum concentration of p-tau396 in PSP (*n* = 13), PD (*n* = 13) and HC (*n* = 12). Data are summarized as box plots. Ranges are depicted as vertical lines while median, 25th percentile and 75th percentile are depicted as middle, lower and upper lines, respectively. Data were analyzed by ANOVA followed by Turkey’s LSD post hoc test. PSP = progressive supranuclear palsy; PD = Parkinson’s disease; HC = healthy control.

Regarding the investigated tau-based biomarkers, only p-tau396 showed detectable levels in serum, while all other tau species (t-tau, 4R tau, tau aggregates, p-tau202 and p-tau231) were below the limit of detection in all samples from patients and controls. Serum levels of p-tau396 were significantly higher in both PD and PSP compared to HC, with no differences between these two patient groups (Table 1 and Figure 1).

### 3.2. Correlation Analysis

**Figure 2:**
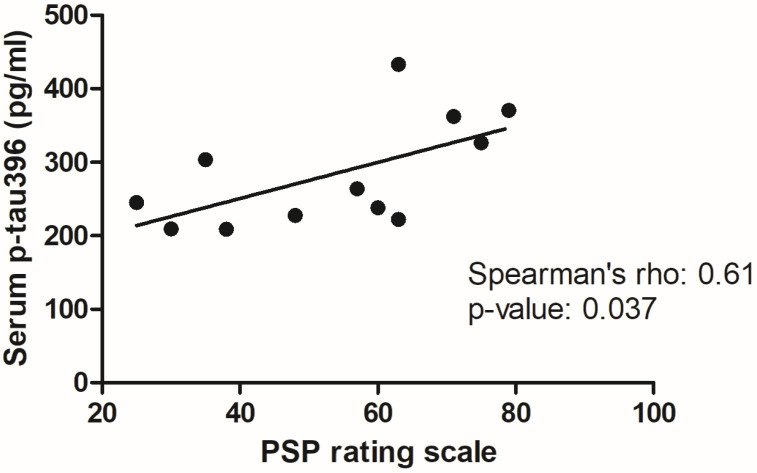
Correlation between serum p-tau396 levels and PSP Rating Scale in PSP patients. The analysis was performed by Spearman’s correlation test, and the obtained rho coefficient and *p*-value are reported in the plot.

Spearman’s correlation tests were performed to investigate the possible association of serum concentration of p-tau396 with demographic and clinical features in both PSP and PD patients as well as with age in the HCs. For the association with disease severity, MDS-UPDRS-III and PSP Rating Scale were used for PD and PSP patients, respectively. Notably, serum p-tau396 showed a significant positive correlation with PSP Rating Scale in the PSP group (Table 2 and Figure 2), while no correlations were found in PD and HC groups.

## 4. Discussion

In this study, we demonstrated that p-tau396 is higher in the serum of PSP and PD patients compared with age- and sex-matched control subjects, and that p-tau396 was associated with disease severity in PSP. On the other hand, 4R-tau, t-tau, tau aggregates and other phosphorylated tau species (p-tau202 and p-tau231) were not detectable in serum using ELISA or SIMOA technologies.

The current clinical diagnosis of PSP mainly relies on clinical signs. However, early symptoms are usually shared with PD, while specific signs such as vertical supranuclear gaze palsy or slowness of vertical saccades, can develop quite late during the disease course [6]. Moreover, PSP can occur in distinct subtypes and some of them are nearly indistinguishable from PD in the early disease stages [33]. Collectively, these factors pose an important challenge for a correct diagnosis [5,6], which in turn affects the correct management of patients as well as the proper selection of cohorts for clinical trials. In light of this, intense efforts have been committed to identify biomarkers to be used in clinical practice. Currently, the best peripheral fluid biomarker to distinguish PSP from PD is the light chain of neurofilament (Nf-L) [34,35,36], which however lacks specificity, being elevated also in other neurodegenerative disorders [36,37].

Histopathological differences observed within the brains of PD and PSP patients have suggested that tau protein, which is found to be phosphorylated and aggregated in NFTs in PSP patients [38], could represent a valuable protein to discriminate PSP from PD. Despite the large number of studies assessing the diagnostic potential of phosphorylated tau species for diagnosing Alzheimer’s disease [3], very few studies evaluated these biomarkers in peripheral fluids in PSP, with few data restricted to p-tau181 in plasma [18,19,20,21] and serum [22].

In a previous study, we analyzed p-tau181 in serum for the first time in PSP and PD patients, showing no differences with respect to HC [22]. Here, we expanded our knowledge of serum tau levels by assessing the presence of six different species of tau in this biological fluid. Surprisingly, we were able to detect only p-tau396, while all the other tested species (t-tau, 4R isoform, aggregates, p-tau202 and p-tau231) were undetectable in all our samples from patients and controls. We hypothesize that this lack of signal might be due to the biological properties of the investigated tau species, to the assessed matrix as well as to the technical limitations of the employed assays.

The absence of tau aggregates in a peripheral fluid can be reasonably explained by the fact that such aggregates might not be circulating due to precipitation [1]. On the other hand, the presence of tau aggregates has been recently reported in CSF [39] and in neuronally-derived exosomes [40]. In both the matrices, PSP patients showed higher levels of tau aggregates compared to PD. While tau aggregates in neuronally-derived exosomes could be detected by the same ELISA kit employed here [39], tau aggregates in CSF were quantified by digital single particle counting, whose sensitivity is in the range of femtomolar, and the emitted signals were close to the limit of detection of the assay [41]. This further supports the hypothesis that tau aggregates are scarcely present in biological fluids.

Different from aggregates, the absence of t-tau and 4R isoform is counterintuitive. Previous research demonstrated that plasma and serum are not interchangeable biofluids for tau measurement [42] and that tau protein can be somewhat altered by thrombin [43]. Therefore, the failure to detect t-tau, detected in plasma in previous studies, might be due to alterations in the tau epitopes recognized by SIMOA kits induced by coagulation enzymes, which are present in plasma but not in serum.

Different from t-tau, which was assessed using SIMOA technology, a hypothesis to explain the lack of several phosphorylated tau species is that their concentration was simply too low to be detected by ELISA. Possibly, the employment of a more sensitive approach such as SIMOA, which exploits fluorescence signal on a dark background to quantify the analyte, and therefore is much more sensitive than ELISA [44], could allow the detection of these tau species. Indeed, SIMOA has been previously applied in the Alzheimer’s field to assess peripheral p-tau231 not only in plasma but also in serum [42,45]. Notably, in both the fluids p-tau231 showed very low concentrations of 5–10 pg/mL, with serum levels slightly lower compared to plasma, reinforcing the hypothesis that serum might not be the best matrix to assess tau species.

Regarding p-tau396, it was detectable in all the samples and showed higher levels in PSP than in HC. This is in line with the hypothesis that tau hypophosphorylation is needed to regulate protein function and that hyperphosphorylation is mainly related to an increased number of phosphorylated tau proteins rather to phosphorylation of many residues on a single molecule [1]. Of note, we detected a strong correlation between p-tau396 serum concentration and disease severity in PSP patients. Accordingly, phosphorylation at Ser396 has been observed in PSP brains [23,24,25,26,27,28], particularly in oligomeric seeds, which have been proposed as the main players in tau spreading and PSP pathology [1,2]. Therefore, such a correlation might reflect a higher burden of tau in PSP patients with more severe symptoms. If validated in larger cohorts, ptau396 may be used as an objective biomarker of disease severity in PSP. Currently, the only other serum molecules associated with disease severity in PSP are Nf-L and IL-6 [34,46]. Further research is warranted to compare these proteins and investigate whether their combination could further improve PSP clinical staging and potentially track or predict disease progression in longitudinal studies.

Regarding the possible use of p-tau396 as a diagnostic biomarker for differential diagnosis in parkinsonism, however, unfortunately p-tau396 levels, as for p-tau181 [22], were largely overlapping between PD and PSP, making this protein not useful in this context. A similar lack of efficacy was reported for p-tau181 in differentiating multisystem atrophy from other parkinsonisms [6,11,19], further supporting the concept that tau hyperphosphorylation is a shared feature in neurodegenerative parkinsonian disorders [2].

Overall, there are several new insights coming from the current study. First, p-tau396 was higher in PSP and PD than in control subjects, with similar values in these two neurodegenerative parkinsonian syndromes. Second, p-tau396 was by far the most expressed tau species in the serum, with several other tau species being either absent or present at too low concentrations to be detected by standard ELISA technology. Third, plasma may be a more suitable blood-derived matrix than serum to investigate t-tau protein levels.

Our work has the novelty of first assessing the presence of several tau species in serum and their diagnostic value in distinguishing PSP from PD. Moreover, we carefully selected patients and controls to be matched for age and sex, and balanced the numerosity of the three groups to minimize the effects of potential confounding factors.

On the other hand, this study also shows some limitations. First, as a pilot study, the number of enrolled subjects is limited overall, which highlights the need for confirmation in larger cohorts. Second, our PSP cohort included PSP-RS and PSP-P patients, which may differ for clinical features and evolution [33]. Recent studies showed that these two PSP subtypes displayed different levels of peripheral biomarkers such as glial-derived neurotrophic factor and hepcidin [47,48]; thus, it is possible to hypothesize that different PSP subtypes might have different levels of p-tau396. Our PSP group was largely composed of PSP-RS patients with only two PSP-P patients having p-tau396 values in the same range of PSP-RS patients. Further analysis comparing PSP subtypes was not carried out due to the low number of patients and this may be the object of future research. Third, most of the investigated tau species were not detected in serum, possibly due to the sensitivity limitation of ELISA assays. Finally, some misdiagnosis might have occurred, since diagnosis was only performed on clinical by movement disorder specialists using international criteria [5,6], but pathological confirmation was missing.

## 5. Conclusions

In this work, we identified p-tau396 as a tool for evaluating clinical staging of PSP. Moreover, our study suggests that ELISA technology on serum is not sensitive enough for the investigation of other phosphorylated tau species, suggesting focusing future efforts on ultrasensitive SIMOA technology to assess tau protein levels in easily accessible blood-based samples, or to explore other substrates such as neuronally derived exosomes.

## Figures and Tables

**Table 1:** Demographic, clinical and serum features of PSP and PD patients and HCs.

	PSP (*n* = 13)	PD (*n* = 13)	HC (*n* = 12)	*p*-Value
Sex (F/M)	6/7	6/7	7/5	0.78 ^a^
Age at examination (years)	70.9 ± 6.36	70.8 ± 6.07	71.0 ± 6.00	1.00 ^b^
Disease duration (years)	4.7 ± 2.89	7.2 ± 5.78	-	0.15 ^c^
MDS-UPDRS-III	60.1 ± 15.91	20.3 ± 9.99	-	<0.0001 ^c^
PSP Rating Scale	53.7 ± 18.17	-	-	-
HY Staging Scale	4.1 ± 0.76	2.0 ± 0.80	-	<0.0001 ^d^
Serum p-tau396 (pg/mL)	290.5 ± 74.33	297.0 ± 71.22	226.2 ± 30.30 *°	0.014 ^b^

PSP: progressive supranuclear palsy; PD: Parkinson’s disease; HC: healthy control; MDS-UPDRS-III: MDS-Unified Parkinson’s Disease Rating Scale part III; HY: Hoehn and Yahr; ^a^ Χ^2^-square test; ^b^ ANOVA followed by Tukey’s LSD post hoc test; ^c^ Unpaired Student’s *t*-test; ^d^ Mann−Whitney test; * HC vs. PD (*p*-value: 0.008); ° HC vs. PSP (*p*-value: 0.015). Data are shown as mean ± standard deviation.

**Table 2:** Correlation analysis of p-tau396 with demographic and clinical variables in PSP and PD patients and with age in HC.

			Age	Disease Duration	PSP Rating Scale	MDS- UPDRS-III
PSP (*n* = 13)	Serum p-tau396	Spearman’s rho	0.31	0.10	0.61	-
		*p*-value	0.29	0.74	0.037	-
PD (*n* = 13)	Serum p-tau396	Spearman’s rho	0.50	−0.35	-	−0.15
		*p*-value	0.08	0.24	-	0.65
HC (*n* = 12)	Serum p-tau396	Spearman’s rho	−0.26	-	-	-
		*p*-value	0.41	-	-	-

## Data Availability

Due to privacy restrictions, the data supporting the results of this study are not publicly available and can be reasonably requested from the corresponding author.
